# The complete chloroplast genome sequence of *Elatostema involucratum*

**DOI:** 10.1080/23802359.2026.2732556

**Published:** 2026-09-16

**Authors:** Meng Zhang, Fang Li, Huihui Meng, Yaohong Feng, YunPeng Li, Yi Zhang, Yingxin Sun, Xiangsong Meng, Chen Zhang

**Affiliations:** ^a^ College of Traditional Chinese Medicine, Bozhou University, Bozhou, China; ^b^ Co-constructing Key Laboratory by Province and the Ministry of Science and Technology of Ecological Restoration and Ecosystem Management, College of Chinese Medicinal Material, Jilin Agricultural University, Changchun, China

**Keywords:** *Elatostema involucratum*, chloroplast genome, phylogenetic analysis

## Abstract

*Elatostema involucratum* Franch. & Sav, a perennial herb in the genus *Elatostema* (family Urticaceae), has significant medicinal value. Here, we report the complete chloroplast genome of *E. involucratum* for the first time, assembled using high-throughput sequencing. The chloroplast genome is 150,481 bp in length, with an overall GC content of 36.35%, and exhibits the typical quadripartite structure, comprising a large single-copy (LSC) region of 84,035 bp, a small single-copy (SSC) region of 17,084 bp, and a pair of inverted repeat (IR) regions of 24,681 bp each. A total of 128 genes were annotated, including 85 protein-coding genes, 35 tRNA genes, and 8 rRNA genes, most of which are associated with photosynthesis and self-replication. Phylogenetic analysis revealed that *E. involucratum* is most closely related to *E. stewardii*, followed by *E. suzukii*. This study enriches the chloroplast genomic resources for the genus *Elatostema*, and provides a valuable foundation for further research on its genetic evolution and germplasm development.

## 1. Introduction

*Elatostema involucratum* Franch. & Sav. (1875), commonly known in China as ‘Banbian-san’ or ‘Lengcao’, is a perennial herbaceous species in the genus *Elatostema* (family Urticaceae). The species is characterized by its distinctive foliage, which is arranged in a staircase-like manner. It thrives in cool, moist, and shaded environments, often forming dense clusters on rocks in valleys, forests, or shrublands. In China, *E. involucratum* is widely distributed, particularly in regions south of the Yangtze River, including northeastern Yunnan, Guizhou, Sichuan, southern Anhui, and southern Shanxi (Editorial Committee of Flora of China, 2003).

Medicinally, the whole plant of *E. involucratum* is used in traditional folk remedies, with reported pharmacological activities, such as heat-clearing and detoxification, promotion of blood circulation and stasis resolution, as well as diuretic and anti-edematous effects (Yang et al. 2014; Editorial Committee of Flora of China, 2003). Meanwhile, numerous studies have investigated the bioactive constituents of plants in the genus *Elatostema* (Miyazawa et al. 2009; Huynh et al. 2025).

In parallel, recent phylogenetic studies have increasingly incorporated molecular evidence to clarify evolutionary relationships within the genus. Among these approaches, shallow genome sequencing to obtain complete chloroplast genomes has become particularly prevalent (Tseng et al. 2025b; Yang et al. 2022; Tseng et al. 2019). Despite these advances, the chloroplast genome of *E. involucratum* has not yet been reported.

In this study, we assembled and characterized the complete chloroplast genome of *E. involucratum* using high-throughput sequencing, thereby providing valuable genomic resources for future research on genetic diversity, evolutionary biology, and germplasm development in the genus *Elatostema*.

## 2. Materials and methods

**Figure 1.**
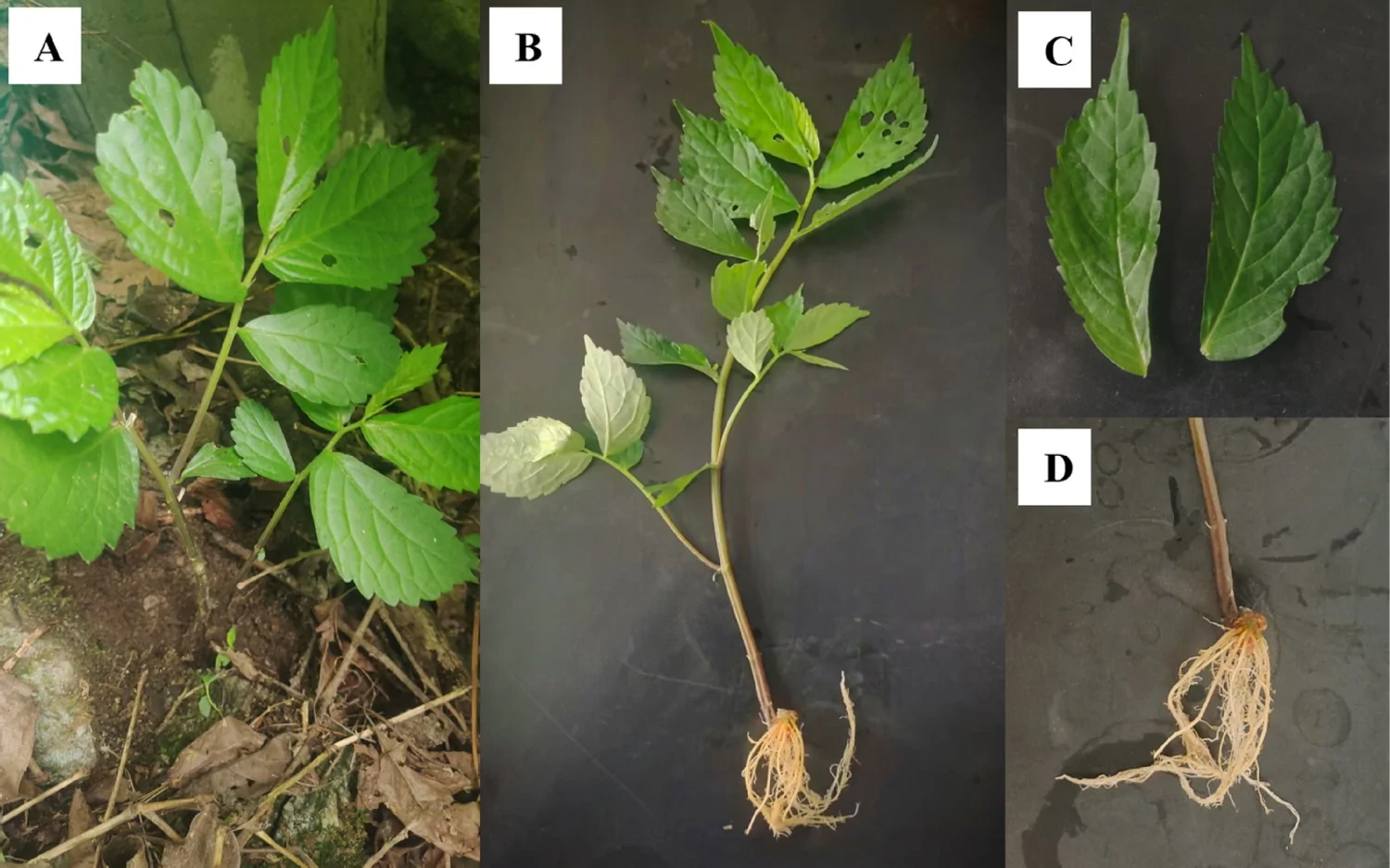
Images of *Elatostema involucratum* (Taken by Meng Zhang). Collection site: Jinzhai County, Anhui Province, China (31.25°N, 115.82°E). Main morphological characteristics: a perennial herb with fleshy stems, unbranched or occasionally with a single branch, glabrous or sparsely pubescent on the upper part. Leaves sessile or nearly sessile, herbaceous, obliquely oblong to slightly falcate, apex abruptly acuminate (margin entire at the tip), base cuneate on the narrow side and rounded on the broad side; venation pinnate, with 5–8 lateral veins on each side. Panels: (A) Habitat; (B) Whole plant; (C) Leaf; (D) Root.

Fresh leaves of *E. involucratum* were collected from Jinzhai County, Lu’an City, Anhui Province, China (31.25°N, 115.82°E). The samples were transported to the laboratory and stored at −80 °C. Habitat photographs of the species were taken during field collection (Figure 1). A voucher specimen was deposited in the Herbarium of Medicinal Plants, Bozhou University (No. 2505018; identified by Xiangsong Meng, e-mail: 1853035272@qq.com). Total genomic DNA was extracted from 150 mg of leaf tissue using the cetyltrimethylammonium bromide (CTAB) method and assessed for quality using a NanoDrop 2000 spectrophotometer (Thermo Fisher Scientific, Waltham, MA, USA) and agarose gel electrophoresis (Gill et al. 2025).

Subsequently, libraries were prepared using a transposase-based kit, amplified with high-fidelity DNA polymerase, and evaluated with a Qubit 4 fluorometer (Thermo Fisher Scientific, Waltham, MA, USA) and an Agilent 2100 Bioanalyzer (Agilent Technologies, Santa Clara, CA, USA). After passing quality control, high-throughput sequencing was performed on the Illumina NovaSeq X Plus platform (Illumina, San Diego, CA, USA) with a 2 × 150 bp paired-end strategy, yielding raw sequencing data.

Raw reads were quality-checked using FastQC v0.11.8, and low-quality sequences and adapters were removed. The filtered reads were assembled into the complete chloroplast genome using GetOrganelle v1.7.7.1 (Jin et al. 2020). The genome was annotated using CPGAVAS2 (http://47.96.249.172:16019/analyzer/home) (Liu et al. 2012), and was then manually curated with reference to the published chloroplast genome of *E. stewardii* Merr. (GenBank accession number: NC_061041). The genome assembly graph was validated using Bandage v0.8.1. The circular chloroplast genome map was generated using Chloroplot (https://irscope.shinyapps.io/Chloroplot/), and gene structures were examined with CPGView (Akrami et al. 2025).

A maximum likelihood (ML) phylogenetic tree was constructed based on the chloroplast genomes of 23 *Elatostema* species, including the newly sequenced *E. involucratum*, with one *Poikilospermum* species used as the outgroup. Protein-coding genes were aligned using MAFFT v7.409 with the G-INS-i strategy (Nakamura et al. 2018). The ML phylogenetic tree was then inferred using IQ-TREE multicore v2.3.6 under the GTR+I + G model with 1,000 bootstrap replicates (Zhang et al. 2025).

## 3. Results

**Figure 2.**
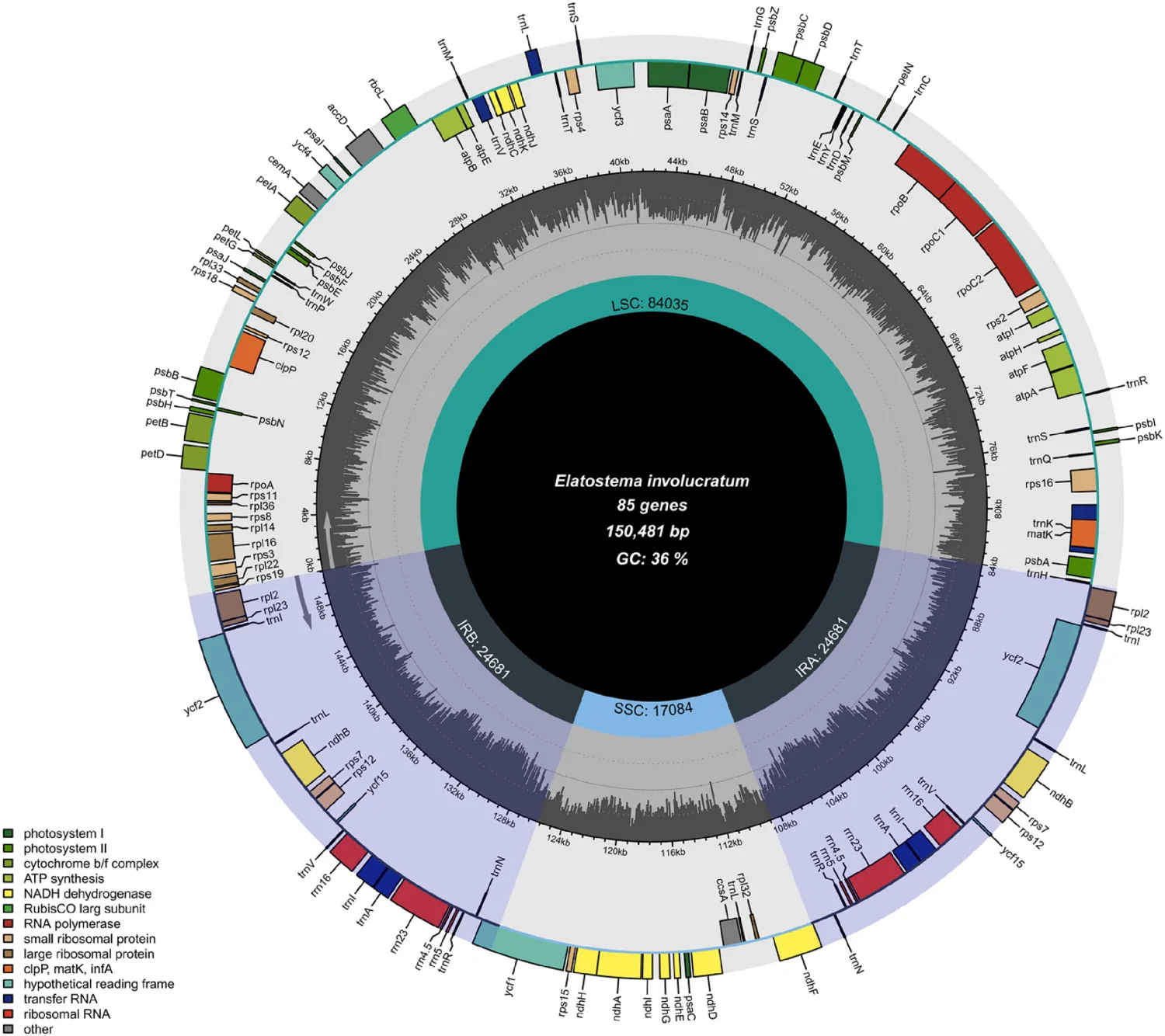
Complete chloroplast genome map of *Elatostema involucratum* generated using Chloroplot. The central panel displays the species name, the number of protein-coding genes (CDS), genome size, and overall GC content. The circular genome map consists of three concentric rings, shown from the innermost to the outermost: the first ring represents the typical quadripartite structure of the chloroplast genome, including the large single-copy (LSC) region, small single-copy (SSC) region, and two inverted repeat regions (IRA and IRB), with the IRs highlighted in blue; the second ring depicts the GC content across the genome; and the third ring shows the annotated genes, color-coded according to functional categories. Arrows on both sides of the third ring indicate transcriptional directions, and the legend in the lower left corner denotes the functional categories of the genes.

We generated 7.11 Gb of high-quality sequencing data for the chloroplast genome of *E. involucratum*. These reads were assembled into a complete chloroplast genome with an average sequencing depth of 771.3× (Figure S1). The circular, double-stranded structure of the genome was validated by visualizing the assembly graph using Bandage, which confirmed the reliability of the assembly (Figure S2). The chloroplast genome exhibits the typical quadripartite structure, with a total length of 150,481 bp and an overall GC content of 36.35%. It comprises a large single-copy (LSC) region of 84,035 bp (GC content: 33.76%), a small single-copy (SSC) region of 17,084 bp (GC content 29.87%), and a pair of inverted repeat (IR) regions of 24,681 bp each (GC content: 42.99%), respectively (Figure 2).

The chloroplast genome of *E. involucratum* encodes a total of 128 genes, including 85 protein-coding genes, 35 tRNA genes, and 8 rRNA genes (Table S1). Most of these genes are primarily involved in photosynthesis and self-replication. In addition, 13 cis-splicing genes (*rpl*16, *pet*D, *pet*B, *clp*P, *ycf*3, *rpo*C1, *atp*F*, rps*16*, rpl*2(2)*, ndh*B(2)*, ndh*A) (Figure S3) and 2 trans-splicing genes (*rps*12(2)) (Figure S4) were identified. Among them, 11 genes (*rpl*16, *pet*D, *pet*B, *rpo*C1, *atp*F*, rps*16*, rpl*2(2)*, ndh*B(2)*, ndh*A) contain a single intron, whereas 4 genes (*clp*P, *ycf*3, *rps*12(2)) contain two introns. The *rps*12 gene has three unique exons, two of which are duplicated in the inverted repeat (IR) regions (Figure S4).

**Figure 3.**
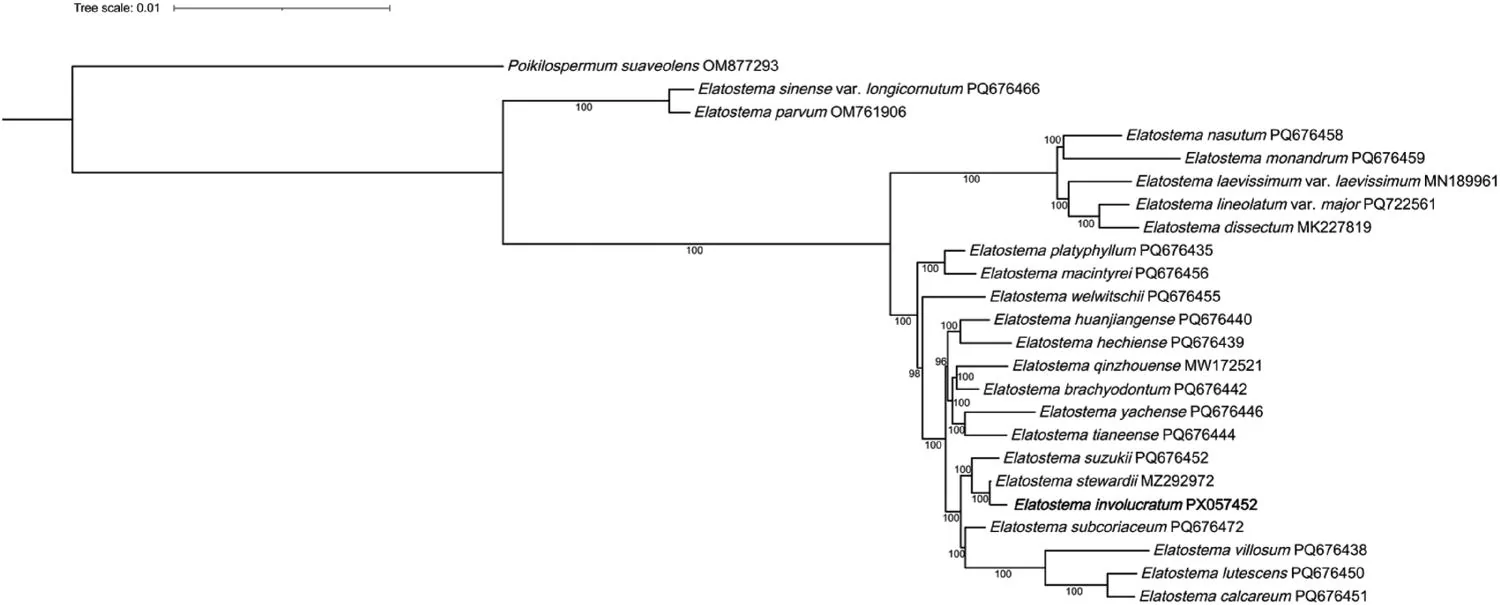
A maximum likelihood phylogenetic tree based on the chloroplast genomes of 23 *Elatostema* species, including *E. involucratum* sequenced in this study, and one *Poikilospermum* species used as the outgroup. Circle sizes on the branches indicate bootstrap support values calculated from 1,000 rapid bootstrap replicates. The scale bar represents the number of nucleotide substitutions per site. The species sequenced in this study (*E. involucratum*) is shown in bold. The chloroplast genome used in this analysis are as follows: *E. sinense* var *longicornutum* (PQ676466)(Tseng et al. 2025b), *E. parvum* (OM761906) (Ogoma et al. 2022), *E. nasutum* (PQ676458) (Tseng et al. 2025b), *E. monandrum* (PQ676459) (Tseng et al. 2025b), *E. laevissimum* var *laevissimum* (MN189961) (Wang et al. 2020), *E. lineolatum* var *major* (PQ722561), *E. dissectum* (MK227819) (Fu et al. 2019), *E. platyphyllum* (PQ676435) (Tseng et al. 2025b), *E. macintyrei* (PQ676456) (Tseng et al. 2025b), *E. welwitschii* (PQ676455) (Tseng et al. 2025b), *E. huanjiangense* (PQ676440) (Tseng et al. 2025b), *E. hechiense* (PQ676439) (Tseng et al. 2025b), *E. qinzhouense* (MW172521) (Fu et al. 2021), *E. brachyodontum* (PQ676442) (Tseng et al. 2025b), *E. yachense* (PQ676446) (Tseng et al. 2025b), *E. tianeense* (PQ676444) (Tseng et al. 2025b), *E. suzukii* (PQ676452) (Tseng et al. 2025), *E. stewardii* (MZ292972) (Yang et al. 2022), *E. subcoriaceum* (PQ676472) (Tseng et al. 2025b), *E. villosum* (PQ676438) (Tseng et al. 2025b), *E. lutescens* (PQ676450) (Tseng et al. 2025b), *E. calcareum* (PQ676451) (Tseng et al. 2025b), *E. involucratum* (PX057452) (the study), *P. suaveolens* (OM877293) (Ogoma et al. 2022).

To investigate evolutionary relationships, a phylogenetic tree was constructed using the chloroplast genomes of 24 Urticaceae species, with *Poikilospermum suaveolens* designated as the outgroup. The remaining 21 *Elatostema* species were grouped in a single clade. Within which *E. involucratum* was most closely related to *E. stewardii*, followed by *E. suzukii* (Figure 3).

## 4. Discussion and conclusion

In this study, we generated the complete chloroplast genome sequence of *E. involucratum* for the first time and systematically characterized its genomic features and phylogenetic position. The complete chloroplast genome was 150,481 bp in length, with an overall GC content of 36.35% (Figure 2), and contained 128 genes, comprising 45 photosynthesis-related genes, 72 genes involved in self-replication and genetic information processing, and 11 other protein-coding genes (Table S1). The genome size, nucleotide composition, and gene composition were highly consistent with those reported for other species of *Elatostema* (Tseng et al. 2025b; Zheng et al. 2017) and conformed to the typical organization of angiosperm chloroplast genomes (Luo et al. 2024; Li et al. 2025; Madayag et al. 2022), supporting the conserved nature of chloroplast genome structure within the genus. Phylogenetic analysis supported a sister relationship between *E. involucratum* and *E. stewardii* (Figure 3), consistent with previous findings suggesting the relatively early divergence of *E. sinense* var. *longicornutum* (Tseng et al. 2025a), thereby providing additional molecular evidence for the phylogenetic position of *E. involucratum* within *Elatostema*.

Compared with previous studies, our results not only expand the available chloroplast genomic resources for the genus but also provide additional molecular evidence for elucidating interspecific relationships within *Elatostema*. The complete chloroplast genome obtained in this study can serve as a reference sequence for molecular identification of closely related *Elatostema* species and for the selection of chloroplast DNA barcode regions, and can also facilitate comparative genomics and the development of molecular markers within the genus, thereby providing valuable genomic resources for germplasm identification and phylogenetic studies. However, the chloroplast genome only reflects the maternally inherited evolutionary history, and the above phylogenetic relationships still need to be further validated by broader taxonomic sampling and multi-genomic data. In future studies, we will expand taxonomic and geographical sampling coverage, integrate nuclear and mitochondrial genomic data to construct a comprehensive genus-level phylogenetic framework, and combine morphological traits with environmental factors to further investigate the mechanisms underlying adaptive evolution in *Elatostema*. Overall, this study provides valuable genomic resources for evolutionary studies of *Elatostema* and establishes a foundation for future integrative analyses across multiple data types.

## Supplementary Material

SMfigures.docx

SMtable.xlsx

## Data Availability

The genomic data supporting the results of this study are publicly available in GenBank of NCBI (https://www.ncbi.nlm.nih.gov/) under accession number PX057452. The associated BioProject, SRA, and BioSample numbers are PRJNA1279342, SRR34060184, and SAMN49478367, respectively.
