# Supplementary material for: The complete chloroplast genome sequence of Elatostema involucratum
Source: Mitochondrial DNA B Resour. 2026 Sep 16;11(10):1162–6. doi: 10.1080/23802359.2026.2732556 (PMC13587581; doi:10.1080/23802359.2026.2732556)
Supplement: Supplementary file 1 [file TMDN_A_2732556_SM6821.docx]

**Figures**

Figure S1 Depth distribution of chloroplast genome sequencing of *Elatostema involucratum*. (The horizontal axis indicates the genome position and the vertical axis indicates the sequencing depth)

Figure S2 Circular structure of chloroplast genome of *Elatostema involucratum*.

Figure S3 Schematic diagram of cis-splicing genes in chloroplast genome of *Elatostema involucratum* (Exons and introns are displayed in black and white respectively; The arrow indicates the translation direction of the gene)

Figure S4 Schematic diagram of trans-splicing gene in chloroplast genome of *Elatostema involucratum*.
